# Advancement of animal and poultry nutrition: Harnessing the power of CRISPR-Cas genome editing technology

**DOI:** 10.5455/javar.2024.k798

**Published:** 2024-06-21

**Authors:** Mahbuba Akther Mishu, Sabuj Kanti Nath, M. Sohidullah, Md. Taslim Hossain

**Affiliations:** 1 Department of Agricultural Finance, Co-operatives and Banking, Khulna Agricultural University, Khulna, Bangladesh; 2 Department of Animal Nutrition, Khulna Agricultural University, Khulna, Bangladesh; 3 Department of Microbiology and Public Health, Khulna Agricultural University, Khulna, Bangladesh

**Keywords:** CRISPR-Cas, gene, nutrition, poultry, welfare

## Abstract

CRISPR-associated proteins and clustered regularly interspaced short palindromic repeats (CRISPR-Cas) technology has emerged as a groundbreaking advancement in animal and poultry nutrition to improve feed conversion efficiency, enhance disease resistance, and improve the nutritional quality of animal products. Despite significant advancements, there is a research gap in the systematic understanding and comprehensive use of the CRISPR-Cas method in animal and poultry nutrition. The purpose of this study is to elucidate the latest advancements in animal and poultry nutrition through CRISPR-Cas genome editing technology, focusing on gene manipulation in metabolism, immunity, and growth. Following preferred reporting items in meta-analysis and systematic reviews guidelines, we conducted a systematic search using several databases, including Scopus, PubMed, and Web of Science, until May 2024, and finally, we included a total of 108 articles in this study. This article explores the use of the CRISPR-Cas system in the advancement of feed additives like probiotics and enzymes, which could reduce the use of antibiotics in animal production. Furthermore, the article discusses ethical and regulatory issues related to gene editing in animal and poultry nutrition, including concerns about animal welfare, food safety, and environmental impacts. Overall, the CRISPR-Cas system holds substantial promise to overcome the challenges in modern animal agriculture. By enriching the nutritional quality of animal products, increasing disease resistance, and improving feed efficiency, it offers sustainable and cost-effective solutions that can revolutionize animal and poultry nutrition.

## Introduction

Considering the growing worldwide demand for sustainable farming to fulfill the sustainable development goals (SDGs) and ensure environmental sustainability, a discussion concerning the effective uses of CRISPR-associated proteins and clustered regularly interspaced short palindromic repeats (CRISPR-Cas) technology in animal and, poultry nutrition is crucial. By investigating the possible uses of the CRISPR-Cas method in advancing ecological sustainability and the SDGs in the context of animal agriculture, this review’s objective is to bridge this gap. By analyzing the complex interactions between gene editing and sustainable food production, this study aims to bring out the groundbreaking promise of the CRISPR-Cas method in overcoming major issues currently facing animal agriculture.

The CRISPR-Cas method is a revolutionary skill that permits specific and efficient editing of genetic information. The abbreviations for CRISPR-Cas are “Clustered Regularly Interspaced Short Palindromic Repeats and CRISPR-associated proteins.” Its mechanism enables bacteria to protect themselves from viruses by cutting and deactivating viral Deoxyribonucleic acid (DNA) sequences. In the areas of agriculture, biotechnology, and medicine, CRISPR-Cas has various potential applications. The CRISPR-Cas method has been designed based on innate defense mechanisms against viral infection of bacteria and archaea, where it works as an adaptive immunity [1,2]. This method comprises three main constituents: (1) the “CRISPR array”, which contains the specific guide Ribonucleic acids (RNAs) that target the DNA sequence of interest; (2) the Cas (CRISPR-associated) proteins, that cleave the DNA at the selected site, and (3) the repair techniques of the cell, which then repair the cut site in one of two ways by using the host cell’s natural DNA repair systems: non-homologous end joining or homology-directed repair (HDR) [3]. The CRISPR-Cas system functions by attaching a guide RNA molecule that matches a certain DNA sequence to the target DNA. Then, the Cas enzyme cuts and modifies the target DNA sequence as needed [3].

The CRISPR-Cas system has an extensive range of applications, from basic research to clinical use. In addition to being used to treat human diseases such as sickle cell anemia, beta-thalassemia [4], and cystic fibrosis by correcting the underlying genetic mutations, the CRISPR-Cas method has been adapted for use in genome editing, allowing researchers to make precise changes to DNA sequences in a wide range of organisms, including animals and plants [5,6]. Although this innovation is being applied to address disease resistance, it is still in the early stages of trial. At this stage, the results have shown significant advancements, suggesting that CRISPR-Cas is promising to transform animal husbandry. Nevertheless, these attributes still need to be thoroughly optimized and corrected. To fully realize the potential of CRISPR-Cas technology and provide long-term, practical solutions for animal and poultry nutrition, further research and experiments are needed to get over the current barriers.

In animal and poultry nutrition, the CRISPR-Cas method is a groundbreaking technology in genome editing, gene therapy, epigenetic modification, and drug delivery within the genome of the animal [7,8]. Its application also involves the creation of genetically adapted crops that are more resistant to pests and diseases and have high nutritional value [9]. Moreover, compared to existing gene-editing instruments, this technique is far more user-friendly, affordable, and highly efficient [10]. For example, CRISPR-Cas 9 is an affordable method of treating avian viral infections in poultry by modifying the host or virus’s DNA [11], and it has a therapeutic role in neurological disorders [12]. Moreover, CRISPR-Cas 9 has more potential in various aspects of diabetes research [13] and the prevention and treatment of Alzheimer’s disease [14]. Additionally, therapeutic uses of this technology include the treatment of congenital heart disease, the prevention of ischemia-reperfusion injury, hyperlipidemias, and arrhythmogenic cardiomyopathies [15]. To control *Eimeria tenella* infection, CRISPR-Cas9 was used to construct a mCherry-GCS1 fusion in *E. tenella* to improve understanding of its transmission and aid the development of gametocidal drugs [16]. Besides, gene editing can be utilized to induce genome modifications that increase tolerance to high temperatures, high humidity, and other extreme conditions in poultry [17].

Furthermore, it is possible to easily design the guide RNA and synthesize it to target any desired sequence, and the Cas nuclease can then be used to cut and edit the targeted gene [18]. This precision allows researchers to do more accurate gene editing, which reduces the risk of unintended consequences. CRISPR-Cas is crucial for improving feed efficiency, disease resistance, and the nutritional quality of products in animal and poultry nutrition. It permits functional feed additives, preserves genetic diversity, and demands ethical and regulatory considerations [19,20–21].

There is a critical research gap in understanding the comprehensive applications of the CRISPR-Cas method in animal and poultry nutrition. Besides, its potential benefits and challenges for sustainable livestock production are still unclear. There are also significant research gaps that include investigating the endless consequences of CRISPR-Cas modifications on animal health, behavior, and reproductive capabilities, as well as understanding the implications of gene editing on genetic diversity and breeding strategies. Additionally, exploring the efficiency of CRISPR-Cas delivery systems and addressing safety and regulatory considerations will be crucial in ensuring responsible and sustainable implementation. Moreover, consumer perception and acceptance of CRISPR-Cas-modified products, as well as conducting comparative analyses with other nutritional strategies, are critical to framing the future of this technology in the livestock and poultry industries. Modern agriculture is facing some urgent issues such as enhancing food security, reducing environmental impact, and promoting animal welfare, and those could be resolved by applying CRISPR-Cas technology. To meet the increasing demand for global food production while maintaining the welfare of both animals and consumers, it is necessary to understand the scope and implications of CRISPR-Cas applications in this context. Research endeavors in the future have the potential to create new opportunities and overcome current restrictions, ranging from investigating novel genetic targets to addressing ethical and regulatory problems. This article highlights the advancement of CRISPR-Cas in animal and poultry nutrition, focusing on improved feed efficiency, disease resistance, and gene manipulation. It also explores functional feed additives’ potential and addresses ethical and regulatory concerns.

## Materials and Methods

### Search strategy

**Figure 1.**
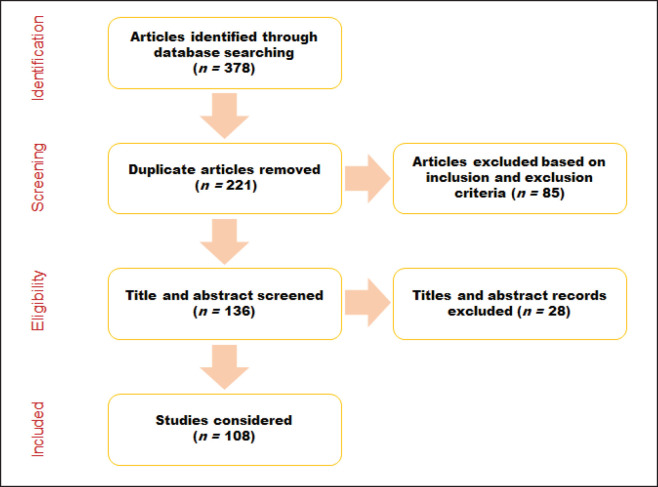
PRISMA diagram of the study selection procedure.

We executed a thorough search technique to identify related articles from reputable scientific databases such as Web of Science, Pun Med, and Scopus. The preferred reporting items in meta-analysis and systematic reviews (PRISMA) framework was followed in the course of this study [22]. Boolean operators were employed to refine the search and extract relevant articles throughout the July 2012–May 2024 timeframe to ensure the inclusion of current and relevant articles. We resolved any discrepancies in the selection process through discussion. Subsequently, 108 articles were chosen for whole-text review (Fig. 1).

### Addition and deletion criteria

Studies that specifically addressed the utilization of CRISPR-Cas tools in animal and poultry nutrition were the focus of the addition criterion. Articles that satisfied the following criteria were accepted for inclusion: they had to address the application of CRISPR-Cas gene-editing techniques to improve feed efficiency, nutrition, or other pertinent aspects in livestock or poultry, and they had to be peer-reviewed and published in English within the allocated time. The effects of genetic alterations on health, production, or the quality of the products in different animal species, such as cattle and poultry, also have to be included in these articles. Exclusion criteria were used to filter out irrelevant research. These included editorials, conference abstracts, publications that were not subjected to peer review, and reviews that lacked primary data. Research without pertinent results or unrelated to CRISPR-Cas uses in animal and poultry nutrition was also excluded.

### Data analysis

Because of the variability in the research design, animal species, and outcomes, a meta-analysis was not practically possible. Instead, to summarize and interpret the findings of the added studies, a narrative synthesis approach was employed. Based on CRISPR-Cas applications, animal or poultry species, and the impact on nutrition, feed efficiency, and animal health, themes and trends were identified, and results were organized.

## Results and Discussion

### How the CRISPR-Cas method works

**Figure 2.**
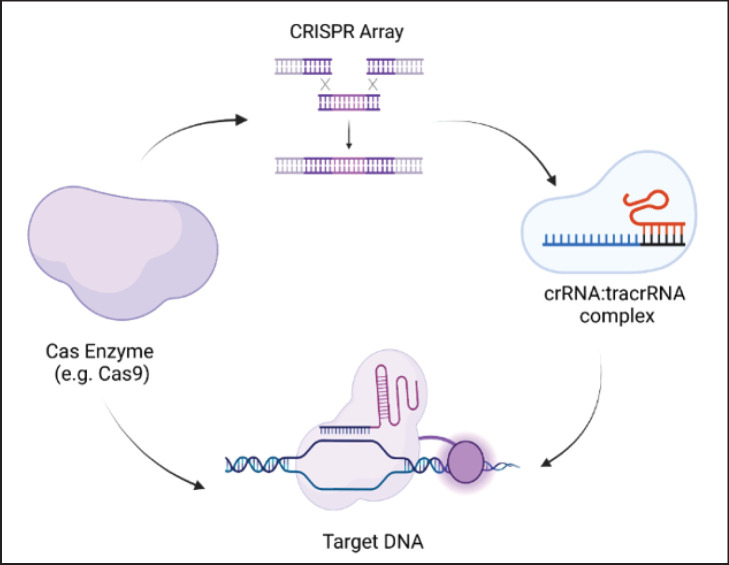
Overview of CRISPR-Cas mechanism.

CRISPR is a short, repeating sequence of DNA existing in the bacterial and prokaryotic genomes, while CRISPR-associated (Cas) proteins are enzymes that are capable of recognizing and cutting DNA at specific locations within the genome [23]. This system employs a guide RNA (gRNA) that is designed to bind to a target sequence of DNA in the genome for gene editing. The gRNA guides the Cas enzyme that can induce a double standard break (DSB) at the target site, triggering the natural DNA-repairing mechanism of the cell (3). DSB can lead to insertions, deletions, or precise gene editing after repair [24]. This technique has vast implementations in diverse fields, involving medicine, agriculture, and biotechnology (Fig. 2).

### Practical implications of the CRISPR-Cas method for animal nutrition

The major practical uses of CRISPR-Cas technology in animal and poultry nutrition are probiotics and enzymes. Another is the development of genetically modified feed additives. These modifications could reduce the requirement for antibiotics in animal production systems, enhance nutrition utilization, and promote digestive health [25]. For instance, researchers have effectively created probiotic strains using the CRISPR-Cas method to enhance nutritional absorption and decrease disease invasion in animals [26]. Besides, researchers could potentially be able to breed livestock with higher feed conversion efficiency, disease resistance, and meat quality qualities by focusing on genes associated with metabolism, immunity, and growth [27], [28]. For example, according to a recent study, it is possible to target certain genes in pigs using CRISPR-Cas technology to boost the production of lean meat and muscle growth [27,29].

1. Using CRISPR-Cas to enhance feed efficiency

Researchers can introduce specific genetic modifications into the candidate genes to enhance their function and, in turn, improve feed efficiency by using CRISPR-Cas technology. For instance, a study showed that introducing a specific genetic modification into the growth hormone receptor gene in pigs by CRISPR-Cas resulted in increased muscle mass and improved feed efficiency [30]. In a different study, the Adipocyte-specific fatty acid-binding protein gene in pigs was genetically modified using CRISPR-Cas, which increased intramuscular fat content and enhanced feed efficiency [31]. Furthermore, gene alterations have been introduced into livestock’s digestive systems using CRISPR-Cas. For example, a study [32] in bovines exhibited that introducing genetic modification into the TLR4 gene by CRISPR-Cas improved the efficiency of the immune response of cattle to pathogen challenges, resulting in enhanced mammary epithelial cells in cows (Table 1).

2. Improving nutrient utilization in animals

By modifying genes involved in nutrient metabolism and absorption, the CRISPR-Cas system can improve nutrient utilization in animals. For example, using CRISPR/Cas9 technology, the myostatin (Mstn) in rabbits and goats was knocked out to examine the impact on skeletal muscle mass and meat output in animals. The results showed the possibility of precise gene editing to enhance the production of meat in these animals [33]. Moreover, research revealed that a combination of CRISPR/Cas9 technology and microinjection has been successfully created for producing enhanced melatonin-enriched milk in sheep [34]. In addition, to create high-value carotenoids with possible protection against age-linked macular degeneration, research demonstrated the useful commercial applicability of CRISPR-Cas9 ribonucleoprotein-produced microalgal mutants in algal biotechnology [59] (Table 1).

Researchers have altered the genomes of cows using CRISPR/Cas to boost the fatty acid metabolism-related enzyme lipoprotein lipase output. The absorption and metabolism of fatty acids can be improved by increasing the production of lipoprotein lipase, which leads to better nutrient utilization efficiency in cows [60]. Moreover, CRISPR-Cas can also be used to develop animals that are resistant to diseases that can impact nutrient absorption and metabolism. For instance, African swine fever (ASF) in pigs can cause high mortality rates, diarrhea, and reduced feed intake, leading to poor nutrient absorption and growth. Researchers have used CRISPR-Cas to generate pigs that are resistant to ASF by deleting a gene that the virus requires to replicate [61] (Table 1).

**Table 1.** Data analysis—narrative synthesis.

Application of CRISPR-Cas	Species of animals/ poultry	Key results	Summary of results	References
Improved feed efficiency through gene editing	Cattle	• Higher growth rates • Increased feed conversion efficiency	Cattle that had undergone CRISPR editing outperformed control groups in terms of demonstrating the potential of gene editing to raise the production of animals.	[35–37]
Biomedicine reasons	Large animals and poultry	• Enhance livestock production efficiency. • Promote animal welfare and health, • Decrease environmental impact and • Improve pest control	There are financial and technical difficulties. Although it may improve animal output and the availability of food, ethical and environmental issues need to be considered in the CRISPR era.	[38,39]
Raising the demand for animal-based food products globally while reducing its negative environmental effects	Cattle, pig, sheep and other livestock	• Improves animal welfare and performance • Potential to lead to sustainable livestock farming with the right regulations	Enhancing livestock productivity and welfare with CRISPR-Cas genome editing is a sustainable approach. New reproductive technologies make it possible to use them on-farm, potentially expediting genetic advancements for a sustainable future in animal farming.	[37,40,41]
Gene editing to enhance the quality of poultry meat	Broiler	• Omega-3 fatty acid concentrations higher in meat • Improved poultry meat quality	The meat from CRISPR-edited chickens contained larger concentrations of advantageous omega-3 fatty acids, leading to healthier poultry products that satisfy customer expectations for nutrient-rich alternatives.	[35,42]
Improved muscle growth with gene knockout	Pig	• Improved muscle mass • Increased in carcass yield	Increased muscle mass in CRISPR-modified pigs due to gene deletion suggests the possibility of greater carcass yield and meat production efficiency, which could have a positive effect on the swine sector.	[43–45]
Disease resistance with gene editing	Livestock and Poultry	• Increased resistance to typical infections • A decrease in antibiotic use	Improved resistance to common infections in CRISPR-edited turkeys resulted in lower antibiotic consumption, promoting sustainable poultry production methods that advance animal welfare and food safety.	[46,47]
Improved Wool/Fiber production by gene modification	Sheep	• Increased production of wool and fibers • Improved fiber/wool quality	Improved wool/fiber output and quality were seen in CRISPR-edited sheep, indicating possibilities for the wool industry and generating higher-quality fibers for the textile industry.	[48–50]
Gene modification to increase milk production	Goat	• Increased milk production • Improved composition of milk	Goats that had undergone CRISPR editing produced more milk and had better milk composition, suggesting that the dairy industry may be improved and higher-quality milk and dairy products could be produced.	[51–55]
Disease-resistance gene knockout	Buffalo	• Increased resistance to a certain disease	CRISPR-edited buffalo produced more milk and had a better milk composition, indicating that the dairy industry may be enhanced and that higher-quality milk and dairy products may be produced.	[41,56]
Improved reproductive performance by gene editing	Other Species	• Improved reproductive characteristics • A rise in reproductive effectiveness	CRISPR-Cas applications demonstrated encouraging outcomes in improving reproductive performance and efficiency in several additional animal species, opening up new opportunities for improved breeding programs and genetic diversity.	[57,40,58]

3. Targeted modification of genes related to growth and development

Researchers achieved a cutting-edge feat by implementing gene editing to disable the beta-lactoglobulin gene in cattle. Their objective was to provide milk that is hypoallergenic and ideal for all consumers, which was published in Edición génica, 2021. Furthermore, studies showed that the CRISPR/Cas9 system’s precise editing or elimination of avian leukosis virus (ALV) receptor genes is the first step toward the generation of hens immune to the ALV [62]. To increase muscle mass and reduce fat deposition in broiler chickens, the growth hormone gene has been successfully targeted and modified using the CRISPR-Cas system [63].

Similarly, the insulin-like growth factor 1 (IGF1) gene has been targeted to increase pigs’ growth performance and meat quality [64]. Moreover, researchers at Seoul National University created swine double-muscled (with more muscular mass) utilizing CRISPR-Cas technology [65]. Apart from that, according to China’s CRISPR 2019, researchers from the Chinese Academy of Sciences created leaner meat with increased endurance to cold conditions by using CRISPR-Cas technology. In addition, according to Genetic Literacy Project 2019, researchers modified the CD163 protein structure by deleting a part of a pig gene using CRISPR-Cas9 technology, preventing porcine respiratory and reproductive syndrome (PRRS) in the pigs with no symptoms of infection or an immune response to the virus. Furthermore, to enhance the quality of the pork, genes encoding enzymes involved in meat tenderization, such as calpastatin and μ-calpain, have been successfully modified [57]. Moreover, reducing fat deposition and increasing feed efficiency have been achieved by targeting the Stearoyl-CoA desaturase (SCD) gene, which is involved in the synthesis of fatty acids in pigs [66]. The MC4R gene involved in appetite regulation in chickens has been targeted to improve feed efficiency and reduce feed intake [35] (Table 2).

4. Growth-related genes

In pigs and cattle, scientists have successfully used CRISPR-Cas to alter genes associated with muscle growth in animals with increased muscle mass and meat yield [67–69]. Similarly, the modification of genes for fat metabolism in animals has led to reduced fat deposition and improved meat quality [43]. In poultry, the modification of growth-hormone-related genes has led to increased growth rates and body weight [70].

5. Improving animal development

In chickens, the alteration of genes involved in embryonic development has led to improved hatchability [71]. The change in bone development-related genes has also led to improved skeletal health and meat quality in pigs [72]. Besides, a study on the CRISPR/Cas9 method showed that heritable double muscle buttocks in rabbits could be achieved through myostatin mutation, which was useful for producing rabbit meat [73]. Another study showed that a CRISPR/Cas9-mediated knockout of the recombination activating gene 1 (RAG1) created an immunodeficient chicken model, enabling avian-specific immune cell development [74].

### Implications for poultry nutrition

1. Using CRISPR-Cas to enhance immune function in poultry

Recent advancements in gene editing technology, like the CRISPR-Cas system, offer new opportunities for poultry to enhance their immune function and resistance to disease. In chickens, it has been demonstrated that utilizing CRISPR-Cas technology to delete the avian interleukin-6 (IL-6) gene increases their resistance to the avian influenza virus [75]. Similarly, the over-expression of the interferon alpha (IFN-α) gene using CRISPR-Cas in chickens has been shown to enhance their antiviral response and reduce the replication of the infectious bursal disease virus [76]. Moreover, it has been demonstrated that utilizing CRISPR-Cas technology to delete the avian toll-like receptor 7 (TLR7) gene in hens lowers the birds’ vulnerability to the infectious bronchitis virus [77].

2. CRISPR-Cas in feed additives

Another approach to CRISPR-Cas use is to enhance immune function in poultry by developing functional feed additives. At present, the probiotic industry commonly utilizes CRISPR-Cas technology to create precisely engineered probiotic lactobacilli [78–80].

Furthermore, the overexpression of the chicken interleukin-2 (IL-2) gene in *Lactobacillus casei* using CRISPR-Cas technology has been shown to upgrade the growth performance and immune function of broiler chickens [81]. In addition to directly targeting immune-related genes, CRISPR-Cas can also be used for the development of functional feed additives that can enhance immune function. For instance, the gene encoding for the antimicrobial peptide cathelicidin has been edited in chicken embryonic fibroblasts, resulting in increased resistance to *Salmonella enteritidis *[81] (Table 2).

These edited genes could be incorporated into probiotics or other feed additives to increase the immune function of poultry. Using CRISPR-Cas technology in *L. casei* has been shown to promote the growth performance and immune function of broiler chickens [82]. Similarly, *Levilactobacillus brevis *has been genetically modified by CRISPR-Cas technology to enhance the functionality and nutritional value of feed additives [91] (Table 2).

**Table 2.** Use of the CRISPR-Cas system in the animal or poultry involved.

Application	CRISPR-Cas system	Animal/Poultry	Study
Improved growth performance	CRISPR-Cas9	Swine	[83,84]
Increased disease resistance	CRISPR-Cas9	Chicken	[8,85]
Improved feed efficiency	CRISPR-Cas9	Swine	[43,61]
Increased meat quality	CRISPR-Cas9	Swine	[31,43]
Decreased influence on the environment	CRISPR-Cas9	Cattle	[41,58]
Improved reproductive efficiency	CRISPR-Cas9	Swine	[45]
Reduced vulnerability to viral infection	CRISPR-Cas13	Chicken	[86,87]
Improved immunological response	CRISPR-Cas9	Swine	[45,88]
Decreased allergic potential	CRISPR-Cas9	Swine	[56,89]
Increasing milk production	CRISPR-Cas9	Cattle	[39,89,90]

3. CRISPR-Cas technique in enzyme production

The CRISPR-Cas technique can be used to engineer microbial strains that produce enzymes beneficial for digestion in poultry. To improve enzyme production efficiency, researchers can target specific genes in microbial producers of enzymes using the CRISPR-Cas system [92,93].

4. Reducing the environmental impact

The use of CRISPR-Cas offers significant potential for sustainable agriculture to lessen the environmental impact of poultry production. Farmers can lower the environmental footprint of poultry production by improving feed efficiency and reducing waste, leading to better food safety and reduced environmental pollution [94–96].

### Potential challenges and risks

Despite its revolutionary promise, there are limitations and restrictions associated with the broad use of CRISPR-Cas technology. Careful inspection and regulation of gene-edited livestock is required due to ethical considerations about animal welfare, food safety, and environmental effects [97]. Robust safety evaluations and regulatory control are crucial due to concerns about unintentional off-target effects and long-term health repercussions for humans and animals [3].

1. Ethical considerations of gene editing in animals

One of the primary ethical considerations of gene editing is the potential for unintentional effects in animals. During gene editing, off-target effects and unintended mutations may occur, which can cause unpredictable and potentially harmful effects in animals. Therefore, careful evaluation is required for potential risks and benefits during the application of CRISPR-Cas technology in animals and to minimize the risk of unintended consequences [98].

Besides, the effect of gene editing on animal welfare is a crucial ethical concern. While it can improve health and disease resistance, there is a risk of unintended consequences like suffering and reduced fitness. Hence, evaluating impact and ethical considerations is essential [99,100]. Moreover, the utilization of CRISPR-Cas technology in animals also raises ethical concerns regarding human health and safety. The potential health risks connected with consuming genetically modified food products should be carefully evaluated to confirm that they are safe for human consumption [99,101].

To efficiently use CRISPR-Cas technology in animal and poultry nutrition, it is crucial that future veterinarians, technicians, and farmers get an education in this particular field. Incorporating the most recent biotechnological advancements, practical training, and ethical concerns, a comprehensive and updated curriculum is needed [102]. Professionals are equipped to manage the societal ramifications of gene editing through interdisciplinary education, which integrates biological sciences, ethics, and communication [103]. Besides, social media also plays an integral role in providing research updates related to welfare and health, with platforms such as Instagram containing posts, stories, reels, live videos, hashtags, and so on [104]. Additionally, the policy implications are crucial and need to be aligned with recent government policies. Its scope must be feasible for the potential implementation of CRISPR-Cas technology in countries with similar socio-economic orientations for the adoption of innovative technology and fostering global dissemination. Globally, many countries have different legal statuses for CRISPR-edited organisms. For example, the USA and China allow their use under certain conditions, whereas the EU and other countries impose restricted limitations or outright prohibitions. The development and application of CRISPR technologies in animal and poultry feeding are impacted by these diverse legal contexts.

2. Potential unintended consequences and risks of CRISPR-Cas technology

Before the widespread adoption of the CRISPR-Cas technique for various applications, the potential unintended consequences and risks of this technology need to be carefully considered and evaluated.

## Off-target effects

When the Cas enzyme accidentally cuts DNA at unexpected sites, off-target effects occur that lead to unintended mutations. Many studies have shown that, depending on the specific CRISPR-Cas system used, off-target effects can occur and that their frequency can vary [105,106].

## Unintended on-target effects

When the desired genetic modification leads to unintended consequences due to its location in the genome or its interaction with other genes or regulatory elements, these occur [107].

## Unintended consequences of gene drives

Through a population, gene drives can rapidly spread a specific genetic modification. However, regarding the potential unintended consequences for the ecosystem, the practice of gene-drive technology raises concerns [108].

## Conclusion

CRISPR-Cas technology holds significant promise to revolutionize animal and poultry nutrition, offering improved health, reduced environmental impact, and enhanced welfare. However, challenges like ethical concerns and ecological risks must be considered carefully. We can harness the capability of CRISPR-Cas to advance sustainable and ethical practices in animal production by addressing such kinds of challenges through responsible research and application.

## List of Abbreviations

ASF, African swine fever; ALV, African Leucosis virus; CRISPR-Cas, CRISPR-associated proteins and clustered regularly interspaced short palindromic repeats; DSB, Double-stranded break; DNA, Deoxyribonucleic acid; FABP4, Adipocyte-specific fatty acid-binding protein; gRNA, Guid-Ribonuclic acid; PRISMA, Preferred Reporting Items in Meta-Analysis and Systematic Reviews; PRRS, Porcine respiratory and reproductive syndrome; RNA, Ribonucleic acid.
